# The impact of acute beta-hydroxy-beta-methylbutyrate (HMB) ingestion on glucose and insulin kinetics in young and older men

**DOI:** 10.1016/j.jff.2020.104163

**Published:** 2020-10

**Authors:** Philip J.J. Herrod, Nima Gharahdaghi, Supreeth S. Rudrappa, Hannah G. Phillips, Reesha A. Ranat, Edward J.O. Hardy, John A. Rathmacher, Philip J. Atherton, Bethan E. Phillips

**Affiliations:** a MRC-ARUK Centre for Musculoskeletal Ageing Research, University of Nottingham, School of Medicine, Royal Derby Hospital Centre, Derby, UK; b Department of Surgery and Anaesthetics, Royal Derby Hospital, Derby, UK; c Metabolic Technologies Inc., Iowa State University Research Park, Iowa, USA

**Keywords:** HMB, Beta-hydroxy-beta-methylbutyrate, Insulin resistance, Glucose tolerance, Age, Supplements

## Abstract

Insulin resistance (IR) is a key feature in the development of numerous metabolic diseases. The cornerstone for treatment for IR remains diet and exercise, however these have poor rates of adherence. Beta-hydroxy-beta-methylbutyrate (HMB) is a nutraceutical with contentious effects on IR in animal models. The aim of this study was to evaluate the impact of acute HMB on IR in humans during an oral glucose tolerance test (OGTT). Young and older male volunteers underwent two 75 g OGTT with or without 3 g HMB. In young men, HMB significantly reduced the insulin area-under-the-curve (AUC), with no difference in glucose AUC, resulting in a numerical increase in the Cederholm index of insulin sensitivity. In older men, HMB had no effect on insulin or glucose responses. In conclusion, acute HMB may improve IR following a glucose load in young men; however, this does not appear to be sustained into older age.

## Highlights

• Insulin resistance (IR) is key to the development of numerous metabolic diseases.
• HMB is a nutraceutical with contentious effects on IR in animal models.
• The effect of HMB during a glucose challenge on insulin resistance is unknown.
• HMB improved insulin sensitivity to a glucose challenge in young, but not older men.
• Clinical trials are needed to establish the chronic effect of HMB on glycaemic control.

## Graphical abstract

**Figure:**
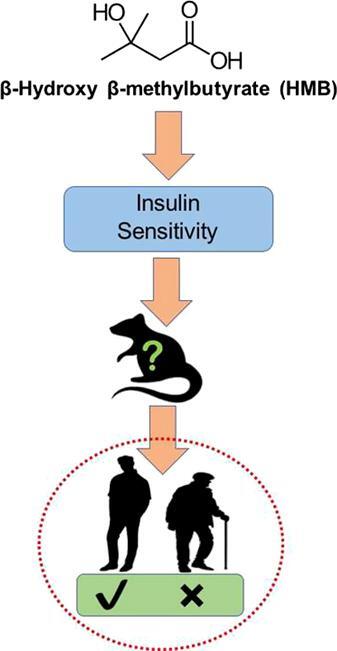

## 1 Introduction

Disordered glucose homeostasis is a key part of the development of a variety of diseases of insulin resistance including type 2 diabetes mellitus and the metabolic syndrome. Type 2 diabetes mellitus is increasing in prevalence across the world (Zheng, Ley, & Hu, 2017) with wide ranging consequences on both healthcare costs and the health of individual patients. Insulin resistance is also well documented to become more prevalent with advancing age (Ryan, 2000), which when taken in the context of an ageing global population further increases its impact.

The cornerstone of treatment for insulin resistance remains diet (Kelly, 2000) and exercise-based (Borghouts & Keizer, 2000) modifications, however, both of these have poor rates of acceptance and adherence (Fappa et al., 2008). Therefore, there remains a need to identify safe and effective nutraceuticals and other nutritional interventions with good adherence to try and combat insulin resistance.

Beta-hydroxy-beta-methylbutyrate (HMB) is a breakdown product of leucine, one of the essential branched chain amino acids, and has several purported anabolic effects. The benefits of dietary supplementation with HMB to enhance anabolic processes have been investigated over many years, with HMB having been demonstrated to increase acute rates of muscle protein synthesis and reduce muscle protein breakdown (Wilkinson et al., 2013). This potential application underlies the main commercial highlight of HMB as a nutraceutical at present. However, studies carried out over longer durations of supplementation have demonstrated conflicting results for changes in body composition and muscle function (Holeček, 2017).

HMB is available commercially in two forms as a nutraceutical; a calcium salt and a free acid (FA) form. Although the calcium salt was previously thought to have a lower bioavailability than the FA, recent evidence has questioned this difference, although not via direct comparison of the two forms (Wilkinson et al., 2018). When given acutely with glucose, the calcium salt form of HMB has previously been shown to have no impact on postprandial glucose or insulin concentrations in younger men (Vukovich et al., 2001), however, a number of chronic supplementation studies in pre-clinical models have demonstrated conflicting effects on insulin sensitivity (Sharawy et al., 2016, Yonamine et al., 2014).

The gold standard tool for assessing insulin sensitivity in humans remains the euglycaemic hyperinsulinaemic clamp (Borai, Livingstone, & Ferns, 2007). However, several indices of insulin sensitivity from an oral glucose tolerance test (OGTT) have been subsequently validated. These indices include those proposed by Matsuda (Matsuda & DeFronzo, 1999) and Cederholm (Cederholm & Wibell, 1990) which reflect combined peripheral and hepatic insulin sensitivity, and mainly peripheral sensitivity, respectively (Patarrão, Lautt, & Macedo, 2014). Higher values for both the Matsuda and Cederholm indices indicate increasing degrees of insulin sensitivity, with the Matsuda index having a strong emphasis on fasting values of insulin and glucose, providing a greater emphasis on the hepatic component of insulin sensitivity, whilst the Cederholm index is more reflective of peripheral insulin sensitivity (Cockcroft, Williams, Jackman, Armstrong, & Barker, 2017).

One potential mechanism by which HMB could affect glucose and insulin kinetics would be through altered peripheral blood flow to muscle (Baron et al., 1995).

Therefore, the aim of this study was to evaluate the acute impact of oral HMB-FA on glucose and insulin homeostasis in young and older men during an OGTT.

## 2 Materials and methods

Institutional research ethics approval (University of Nottingham Faculty of Medicine and Health Sciences Research Ethics Committee) was obtained (A12092016 SoM MS GEM) to recruit 10 healthy young (aged 18–35 y) and 10 healthy older (aged 65–85 y) male subjects by local advertising. Exclusion criteria included extremes of BMI (<18 or > 32 kg/m^2^) and diagnosis of metabolic disorders. The study was registered with clinicaltrials.gov (NCT03018496) and complied with the 1964 Declaration of Helsinki.

After providing written informed consent to participate in the study, all subjects underwent a clinical examination involving measurements of height, weight and resting blood pressure (OMRON M3, Omron Healthcare, UK) and fasting glucose before being enrolled. Each subject performed two, 3-hour 75 g oral glucose tolerance tests (OGTT) (Ivey et al., 2007, Wopereis et al., 2009), spaced 2-weeks apart. During one OGTT, the glucose was administered with 3 g HMB-FA (a dose previously shown to produce a measurable effect on protein metabolism (Wilkinson et al., 2013)), with glucose alone administered during the alternate OGTT. The order in which the subjects completed the two studies was randomized via a computer-generated randomization tool (sealedenvelope.com).

On each study day, subjects presented to the laboratory at 0900 h fasted from 2100 h the preceding evening. On arrival, a 22G retrograde intravenous cannula was inserted into a vein in the dorsum of a hand to facilitate blood sampling (using the hot-hand method (heated to 55 °C) (Liu et al., 1992)). Blood was drawn for real-time quantification of blood glucose concentrations with aliquoted plasma frozen for later quantification of insulin concentrations. Blood glucose concentrations were determined using a near patient glucose analyzer (YSI Life sciences, Ohio, USA). Plasma insulin concentrations were determined using an ultrasensitive enzyme linked immunosorbent assay (ELISA) (Mercodia Ultrasensitive Insulin ELISA, Mercodia, Uppsala, Sweden).

After baseline measures, participants drank 75 g oral dextrose (Myprotein, Northwich, UK) dissolved in 200 mL of water, with or without 3 g HMB-FA gel (Metabolic Technologies Inc., Ames, Iowa, USA) provided immediately after the glucose (within 2 min of the dextrose). Blood samples were drawn at baseline then 15, 30, 45, 60, 80, 100, 120, 140, 160 and 180 min after the dextrose drink.

Leg blood flow was measured as a potential mechanism by which HMB could affect glucose and insulin kinetics. Measurements were made of the common femoral artery of the right leg at three time points during each study visit (baseline, 60 and 120 min after dextrose) using Doppler ultrasound (iU22, Phillips Healthcare, Guildford, UK) and a 9–3 mHz probe. Blood flow was calculated as the product of the artery cross sectional area and the mean blood velocity over 3 cardiac cycles (Phillips, Williams, Atherton, Smith, Hildebrandt, Rankin, & Rennie, 2012).

Area-under-the-curve (AUC) analyses were performed for both glucose and insulin concentrations facilitating calculation of the Cederholm and Matsuda indices of insulin sensitivity, with the homeostatic model of insulin resistance (HOMA-IR) calculated from fasting values (Patarrão et al., 2014). HOMA-IR was calculated using the formula:

$$\frac{\left[Fasting\;insulin(mUL^{-1})\right]\times \left[Fasting\;glucose\left({mmolL}^{-1}\right)\right]}{22.5}$$

β-hydroxy-β-methylbutyric acid (HMB-FA, CAS# 625-08-1; Formula: C5H10O3) was manufactured by TSI Co., Ltd (Shanghai, China) for Metabolic Technologies, Inc. (Ames, IA, USA) and purity was 98% as measured by high performance liquid chromatography (HPLC).

### 2.1 Statistics

All statistical analyses were performed using Graphpad Prism Version 7.02 (California, USA) with data presented as mean (SD). After confirmation of normality, analysis was performed using 2-way ANOVA by age and treatment with Sidak’s multiple comparison post-hoc testing. Significance was set at *p* < 0.05.

## 3 Results

Ten young and ten older subjects were recruited with all subjects completing both OGTT’s (subject characteristics are displayed in Table 1). Definitions for impaired fasting glucose and impaired glucose tolerance were taken from the World Health Organization Guidelines (World Health Organisation, 2006). There was no significant difference in HOMA-IR between the age-groups and no subject from either age-group presented with impaired fasting glucose.

**Table 1:** Subject characteristics (Mean (SD)).

	Young participants(N = 10)	Older participants(N = 10)
Age (years)	22 (4)	72 (3)^1^
Height (cm)	179 (6)	176 (6)
Weight (Kg)	81 (10)	82 (7)
BMI (Kg/m^2^)	25.4 (2.5)	26.5 (2.7)
Systolic blood pressure (mmHg)	129 (8)	143 (19)^2^
Diastolic blood pressure (mmHg)	76 (9)	83 (15)
HOMA-IR	1.2 (0.9)	1.3 (0.6)
Impaired fasting glucose (6.1–6.9 mmol/L) (n)	0	0
Impaired glucose tolerance 120 min sample (>7.8 < 11.1 mmol/L) (n)	0	7

1 p < 0.001 vs. young participants.

2 p < 0.05 vs. young participants.

**Fig. 1:**
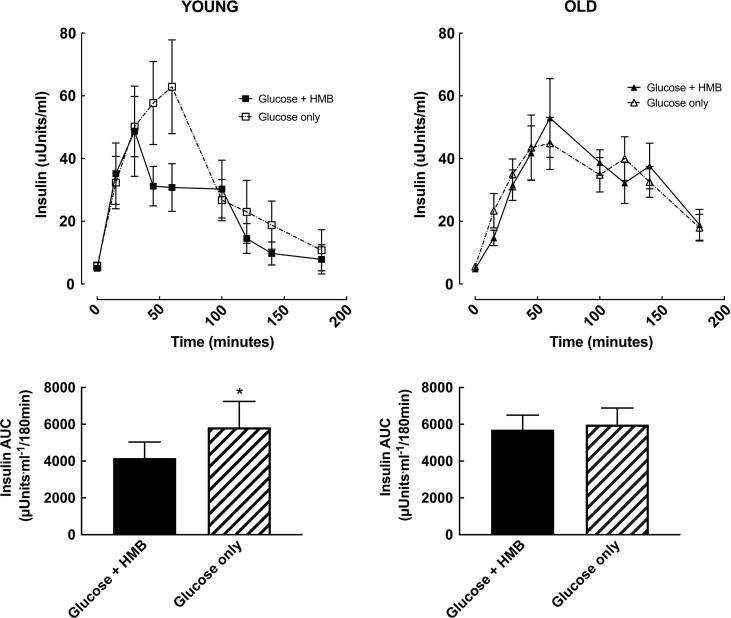
Insulin concentrations and insulin area-under-the-curve (AUC) before (time 0) and 3-hours after a 75 g oral glucose challenge, with or without 3 g HMB, in young (18–35 y, n = 10; left) and older (65–85 y, n = 10; right) men. *= *p* < 0.05 vs. Glucose + HMB.

**Fig. 2:**
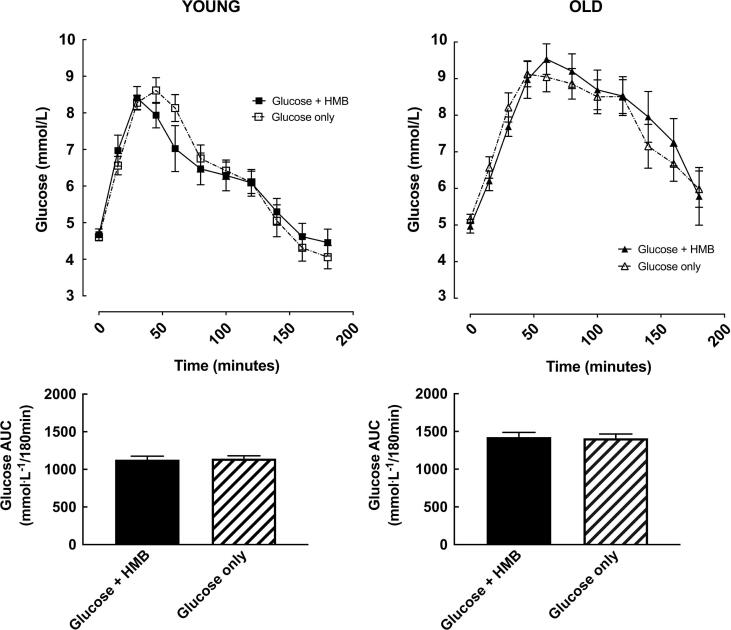
Glucose concentrations and glucose area-under-the-curve (AUC) before (time 0) and 3-hours after a 75 g oral glucose challenge, with or without 3 g HMB, in young (18–35 y, n = 10; left) and older (65–85 y, n = 10; right) men.

**Fig. 3:**
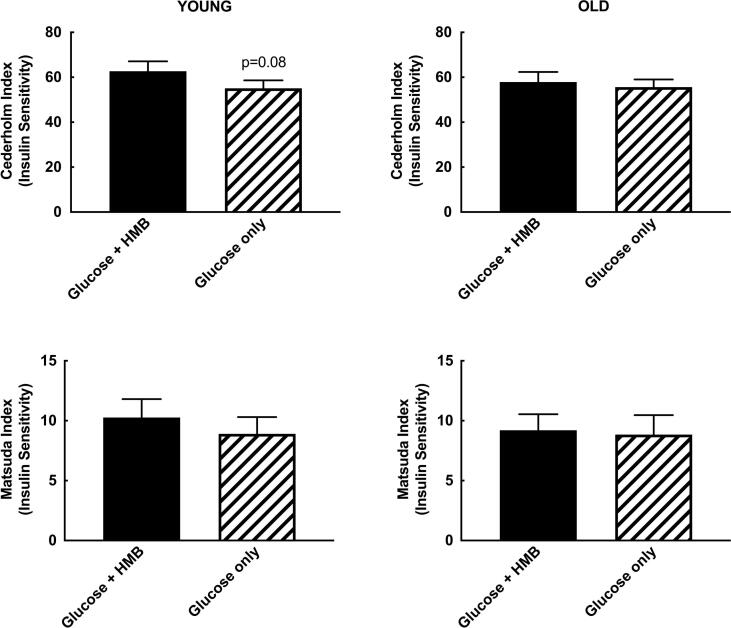
Cederholm and Matsuda indices of insulin sensitivity in young (18–35 y, n = 10; left) and older (65–85 y, n = 10; right) men after a 75 g glucose challenge, with or without 3 g HMB. *p* = 0.08 vs. Glucose + HMB.

There was a main effect of time for insulin and glucose concentrations in both young and older subjects (all p < 0.0001). HMB significantly reduced the insulin AUC (4158 (2753) vs. 5836 (4451) mU/Lx180min; *p* = 0.02) in younger subjects (Fig. 1), with no difference in glucose total AUC (1127 (147) vs. (1142 (122) mmol/Lx180min; *p* = 0.8) (Fig. 2). This led to a numerical increase in the Cederholm index of insulin sensitivity (62.7 (13.9) vs. 55.1 (11.0); *p* = 0.08)). There was no change in the Matsuda index of insulin sensitivity (10.3 (4.9) vs. 8.9 (4.4); *p* = 0.19) (Fig. 3).

In older subjects, HMB had no effect on the insulin (5712 (2488) vs. 5980 (2854) mU/Lx180min; *p =* 0.88) (Fig. 1) or total glucose AUC (1426 (194) vs. (1409 (180) mmol/Lx180min; *p* = 0.84) (Fig. 2), with no resultant difference in either their Cederholm (57.9 (14.2) vs. 55.6 (10.7); *p* = 0.72) or Matsuda index (9.2(4.3) vs. 8.8(5.2); *p* = 0.88) (Fig. 3). There was a main effect of age on total glucose AUC, which was not present for insulin AUC or either index of insulin sensitivity. All of the statistical findings reported for total glucose AUC were also apparent for incremental glucose AUC.

HMB did not alter leg blood flow responses to glucose in either the young or older participants at any time point, although there was a main effect of age at all three time points (*p* < 0.001) (Table 2).

**Table 2:** Leg blood flow (ml/min).

Time	Young			Older		
	HMB	No HMB	p	HMB	No HMB	p
Baseline	510 (148)	440 (147)	0.37	275 (97)	281 (121)	0.99
60 min	478 (168)	534 (180)	0.74	334 (134)	371 (150)	0.79
120 min	572 (183)	518 (148)	0.69	343 (119)	302 (113)	0.81

## 4 Discussion

This study has demonstrated that a single 3 g dose of orally administered HMB-FA can reduce insulin responses to an oral glucose challenge in young men, leading to a suggestion of improved insulin sensitivity. However, it would appear that this effect is not sustained into older age which may limit the potential use of HMB as a nutraceutical for improving insulin sensitivity, as insulin resistance is most prevalent in older age (Ryan, 2000).

As HMB, in both the calcium salt and FA form, has been previously shown to enhance acute anabolic responses (Wilkinson et al., 2013, Wilkinson et al., 2018), that its administration may lead to an acute increase in insulin sensitivity is plausible given the well-established role of insulin in anabolic processes (Wilkes et al., 2009). However, the anabolic effect of HMB has also been demonstrated in older adults (Holeček, 2017), and as such the reason why an insulin sensitizing effect is not apparent with advancing age remains unclear.

In agreement with reports across numerous studies illustrating the positive effect of Leucine on insulin sensitivity (Zhang et al., 2020), our data herein suggests that certain amino acid metabolites may also have the ability to positively modulate glucose metabolism under specific conditions. One potential mechanism that may underlie any improvement in insulin sensitivity could be an increase in leg blood flow facilitating greater peripheral glucose uptake, however this increase was not evident in this study. It may be that as leg blood flow was only measured at two ‘postprandial’ time-points, an enhancement in leg blood flow that occurred at either an earlier or later time-point has been missed.

It is interesting that the results of this study differ from those of Vukovich et al. (Vukovich et al., 2001) who demonstrated that acute HMB supplementation had no effect on plasma insulin or glucose concentrations in young men. Possible reasons for this disparity may include the method of insulin quantification (radioimmunoassay by Vukovich et al., whilst ELISA by ourselves), a reduced sampling frequency in the Vukovich study (large differences were apparent at 45 and 140 min in our study, time points which were not measured by Vukovich et al.,) and/ or the different HMB formulations given (Vukovich et al., used the calcium salt whereas the free acid form may have greater oral bioavailability (Fuller, Sharp, Angus, Baier, & Rathmacher, 2011)).

That HMB elicited a numerical increase in the Cederholm index of insulin sensitivity in our young participants, whilst having no effect on the Matsuda index may indicate that HMB is affecting peripheral rather than hepatic aspects of insulin sensitivity (Gutch, Kumar, Razi, Gupta, & Gupta, 2015). This is contrary to what has previously been demonstrated in a chronic study of HMB in rodents where an improvement in hepatic insulin sensitivity mediated via GLUT-2 was shown (Sharawy et al., 2016). A separate chronic HMB rodent study did however show a potential reduction in GLUT-4 mediated peripheral insulin sensitivity (Yonamine et al., 2014), a finding which may be more in keeping with the results of this study. As such, further investigation of the mechanistic basis behind the differential effects of HMB on acute glucose handling with advancing age should be concentrated on looking at peripheral mechanisms of insulin sensitivity, including a more detailed analysis of vascular responses. In addition, chronic supplementation studies measuring longer-term changes in insulin sensitivity will be required before HMB could be considered as a therapeutic agent for improving insulin sensitivity. Finally, although the results herein suggest that the detrimental impact of HMB on insulin sensitivity seen in some rodent studies (Yonamine et al., 2014) does not translate to humans, further clinical trials are required to confirm this.

A limitation of this study is that, beside subjects being asked to replicate their dietary and physical activity behaviours for the 24-hours before each study visit, no further control or recording of dietary behaviour between the two study visits was employed. However, all subjects were studied after a 12-hour fasting period, likely negating the impact of this limitation. One further limitation of our study was that it recruited male subjects only, limiting the interpretation of our results with regards to female populations.

In summary, acute HMB-FA can significantly reduce plasma insulin concentrations in young men in response to a glucose challenge, without altering blood glucose concentrations, leading towards an improvement in insulin sensitivity. This is not the case in older men. Future studies will be required to assess the impact of chronic supplementation with HMB-FA on insulin sensitivity with a thorough consideration of age (e.g., middle-aged adults) and gender.

Ethics statement

All work reported in this manuscript has been carried out in accordance with the Code of Ethics of the World Medical Associated (Declaration of Helsinki) and was approved by the University of Nottingham Faculty of Medicine and Health Sciences Research Ethics Committee under reference code A12092016 SoM MS GEM.

## CRediT authorship contribution statement

**Philip J.J. Herrod:** Conceptualization, Methodology, Validation, Formal analysis, Investigation, Data curation, Writing - original draft, Writing - review & editing, Visualization. **Nima Gharahdaghi:** Formal analysis, Investigation, Writing - original draft, Writing - review & editing, Visualization. **Supreeth S. Rudrappa:** Investigation, Writing - original draft, Writing - review & editing. **Hannah G. Phillips:** Investigation, Writing - original draft, Writing - review & editing. **Reesha A. Ranat:** Investigation, Writing - original draft, Writing - review & editing. **Edward J.O. Hardy:** Validation, Investigation. **John A. Rathmacher:** Resources, Writing - original draft, Writing - review & editing. **Philip J. Atherton:** Conceptualization, Writing - original draft, Writing - review & editing, Supervision. **Bethan E. Phillips:** Conceptualization, Methodology, Formal analysis, Writing - original draft, Writing - review & editing, Visualization, Supervision.

## Declaration of Competing Interest

The authors declare no conflicts of interest. JAR is an employee of Metabolic Technologies Inc., who supplied the free acid-HMB on a collaborative basis. Metabolic Technologies Inc., has patents pending on HMB-FA, and market HMB to nutrition companies.
